# Improving Influenza HA-Vlps Production in Insect High Five Cells via Adaptive Laboratory Evolution

**DOI:** 10.3390/vaccines8040589

**Published:** 2020-10-07

**Authors:** Ricardo Correia, Bárbara Fernandes, Paula M. Alves, Manuel J.T. Carrondo, António Roldão

**Affiliations:** 1 IBET, Instituto de Biologia Experimental e Tecnológica, Apartado 12, 2780-901 Oeiras, Portugal; rccorreia@ibet.pt (R.C.); bfernandes@ibet.pt (B.F.); marques@ibet.pt (P.M.A.); mjtc@ibet.pt (M.J.T.C.); 2 ITQB NOVA, Instituto de Tecnologia Química e Biológica António Xavier, Universidade Nova de Lisboa, Av. da República, 2780-157 Oeiras, Portugal

**Keywords:** adaptive laboratory evolution, insect High Five cells, baculovirus expression system, influenza HA-VLPs, improved production

## Abstract

The use of non-standard culture conditions has proven efficient to increase cell performance and recombinant protein production in different cell hosts. However, the establishment of high-producing cell populations through adaptive laboratory evolution (ALE) has been poorly explored, in particular for insect cells. In this study, insect High Five cells were successfully adapted to grow at a neutral culture pH (7.0) through ALE for an improved production of influenza hemagglutinin (HA)-displaying virus-like particles (VLPs). A stepwise approach was used for the adaptation process, in which the culture pH gradually increased from standard 6.2 to 7.0 (ΔPh = 0.2–0.3), and cells were maintained at each pH value for 2–3 weeks until a constant growth rate and a cell viability over 95% were observed. These adapted cells enabled an increase in cell-specific HA productivity up to three-fold and volumetric HA titer of up to four-fold as compared to non-adapted cells. Of note, the adaptation process is the element driving increased specific HA productivity as a pH shift alone was inefficient at improving productivities. The production of HA-VLPs in adapted cells was successfully demonstrated at the bioreactor scale. The produced HA-VLPs show the typical size and morphology of influenza VLPs, thus confirming the null impact of the adaptation process and neutral culture pH on the quality of HA-VLPs produced. This work strengthens the potential of ALE as a bioprocess engineering strategy to improve the production of influenza HA-VLPs in insect High Five cells.

## 1. Introduction

Influenza is a major burden to global healthcare systems, often causing pandemics with massive death-tolls [1,2]. Prophylactic vaccination still remains the most efficient approach to reduce influenza disease [3]. Due to the high tendency of influenza viruses to accumulate 3–4 amino acid substitutions per year and to genetically reassort amongst viruses in animal reservoirs [4], vaccine composition needs to be updated and new vaccines need to be formulated, produced and administrated on an annual basis, if not more often in the case of pandemics. Thus, the development of fast, economical and flexible vaccine production platforms to quickly cope with the need for high quantities of vaccine shots has become a worldwide key healthcare priority.

Vaccine manufacturing platforms based on cell culture allow superior process control and flexibility, potentially improving the responsiveness to influenza epidemics or pandemics [5]. Mammalian cells have been extensively explored and proven efficient for the production of influenza vaccines [6,7]. Virus-like particles (VLPs) allow for a high level of antigen display, thus showing high potential to be used as vaccines. The insect cell-baculovirus expression vector system (IC-BEVS), which allows for a fast production of high protein titers, has proven to be efficient to produce complex VLPs [8], including influenza VLPs similar to those produced by mammalian cells [9].

The use of non-standard values of cell culture parameters such as pH, temperature or dissolved oxygen concentration has often been shown to significantly impact the growth performance and recombinant protein production yields in different cell hosts [10,11,12]. Most reports refer to the use of single or multiple shifts in such parameters as effective means to enhance cell performance [13,14]. Alternatively, the use of adaptive laboratory evolution (ALE), i.e., adaptation of cells to efficiently grow under such non-standard culture conditions, through consecutive sub-culturing under these selective pressures allows for the selection of cell populations with an enhanced fitness. Besides improving cell growth [15] by enabling beneficial mutations to arise [16], this process also leads to more efficient energy utilization, the use of non-conventional substrates [17], tolerance to byproducts of interest [18,19], or even the development of thermo-resistant strains [20].

Importantly, ALE has been suggested as an approach to maximize recombinant protein titers in both prokaryotes and animal cells [21,22]. However, it has been poorly explored in insect cells. Recently, adaptation to hypothermic culture conditions has proven efficient to significantly increase the production of HIV1 Gag VLPs using stable insect cell lines [23]. Additionally, ALE has proven to be efficient to increase the production of Chikungunya VLPs by more than 11-fold using IC-BEVS with insect *Sf-21* cells adapted to a higher pH [24]. Thus, we have hypothesized that adaptation to higher culture pH could also have an impact on the production of other VLPs using insect cells.

In this work, High Five insect cells were adapted to grow at neutral pH and their capacity to produce influenza A hemagglutinin-displaying virus-like particles (HA-VLPs) was assessed, aiming at developing a platform for a faster production of influenza vaccine candidates.

## 2. Materials and Methods

### 2.1. Cell Line and Culture Media

High Five insect cells (Invitrogen, kindly provided by Redbiotec AG, Schlieren, Switzerland) were routinely sub-cultured at 0.3–0.5 ×10^6^ cell.mL^−1^ every 2–3 days when cell concentration reached 2–3 ×10^6^ cell.mL^−1^ in serum-free Insect-XPRESS^TM^ medium (Lonza, Basel, Switzerland) (herein mentioned as CM_pH6.2_) using 125–500 mL shake flasks (Corning, New York, NY, USA) with a 10% working volume, and maintained at 27 °C in a shaking incubator (Inova 44R – Eppendorf, Hamburg, Germany) set to 100 RPM and with 2.54 cm shaking diameter.

### 2.2. Adaptation of Insect Cells to Neutral pH

Culture medium containing a 1:1 mixture of Insect-XPRESS^TM^ medium and a chemically defined solution containing 50 mM HEPES, 124 mM Sucrose, 5 mM Glucose, 50 mM NaCl, 20 mM KCl, 3 mM CaCl_2_, 10 mM MgSO_4_ and 0.1% (w/v) Pluronic F-68 [24] was used for the adaptation process of High Five cells to neutral pH (hereon referred to as CM_pH7_). The pH was adjusted to the desired value by adding NaOH 1 M and sterile filtered using a 0.22 µm Stericup (Millipore, Burlington, MA, USA). A stepwise approach was used for the adaptation process, in which culture pH was gradually increased from standard 6.2 to 6.5, 6.8 and finally to 7.0, and cells maintained in each pH value for approximately 2–3 weeks until a constant growth rate and cell viability over 95% were observed. To circumvent medium acidification during cell growth, culture pH was monitored daily using a benchtop probe (Crison, Barcelona, Spain) and adjusted by aseptically adding sterile NaOH 1M at a proportion of 15 µL/pH unit/mL of culture. Master cell banks were prepared prior to each pH increase by re-suspending cells in CryStore CS10 freezing medium (Sigma, St. Louis, MO, USA) and freezing at −80 °C using a Coolcell cell freezing container (Biocision, Larkspur, CA, USA).

### 2.3. Baculovirus Amplification and Storage

Recombinant baculoviruses containing influenza M1 (from A/California/06/2009 H1N1 strain) and HA (from A/Brisbane/59/2007 strain) genes were kindly provided by Redbiotec AG (Schlieren, Switzerland). An amplification of baculovirus stocks was performed as described elsewhere [25]. Briefly, insect *Sf-9* cells (cultivated in Sf-900^TM^ II medium (Gibco, Waltham, MA, USA)) were infected at a concentration of 1 × 10^6^ cell/mL using a multiplicity of infection of 0.1 plate-forming units per viable cell (pfu/cell). When a cell viability of approximately 80% was reached, the supernatant was harvested by centrifugation at 200× *g* and 4 °C for 10 min and centrifugation at 2000× *g* and 4 °C for 20 min. The clarified supernatant was aliquoted appropriately and stored at 4 °C until further use.

### 2.4. Production of HA-Displaying VLPs

VLPs displaying HA were produced in 250 mL shake flasks (10% working volume) and in 0.5 L glass stirred-tank bioreactors.

In shake-flask cultures, cells were cultured in 500 mL shake flasks (Corning, NY, USA) with a 10% working volume in either CM_pH6.2_ (for non-adapted cells) or CM_pH7_ (for adapted cells). Infection experiments were performed in 250 mL shake flasks (Corning, NY, USA), with a 10% working volume, and at different cell concentrations at the time of infection (CCIs; 1 × 10^6^, 2 × 10^6^ and 3 × 10^6^ cell.mL^−1^) and multiplicity of infection (MOI; 0.1, 1 and 10 pfu.cell^−1^). At the time of infection, a complete medium exchange was performed by centrifugation at 200 g at room temperature for 10 min.

Bioreactor runs were operated in computer-controlled BIOSTAT Qplus 0.5 L vessels (Sartorius, Göttingen, Germany) using adapted cells at generation 53 after the establishment of the master cell bank. Culture mixing was achieved by equipping the bioreactor with one Rushton impeller and varying the agitation rate from 70 to 270 rpm. Gas was supplied through a ring sparger at a flow rate of 0.01 vvm, and the percentage of O_2_ in the gas mixture varied between 0 and 100%, to maintain the pO_2_ (partial pressure of oxygen) setpoint at 15% of air saturation. The bioreactor headplate included multiple ports for temperature, pH and pO_2_ probes as well as for additions (i.e., culture medium, cells and baculovirus) and the sampling/harvesting of the cell culture. Culture pH was controlled at 7.0 by adding NaOH 1 M. Bioreactors were operated at a working volume of 0.3 L, inoculated at a cell concentration of 2 × 10^6^ cell.mL^−1^, and infected immediately at an MOI of 1 pfu.cell^−1^.

### 2.5. Purification of HA-Displaying VLPs

Culture bulk from bioreactor runs was harvested and centrifuged at 4 °C, 200 g, for 10 min (for cell removal) and at 4 °C, 2000 g, for 20 min (for further clarification). The clarified supernatant was filtered using a 0.22 µm Stericup (Millipore, Burlington, MA, USA), and HA-VLPs were concentrated/purified using a (scheme 1) polyethylene glycol (PEG) precipitation method followed by size-exclusion chromatography, or (scheme 2) anion-exchange chromatography. In scheme 1, proteins were precipitated overnight with PEG (8.5% w/v) and NaCl (0.3 M). The precipitated proteins were centrifuged at 4500 g and 4 °C for 30 min, pellets were re-suspended in buffer containing HEPES and NaCl, and the concentrated protein content was purified by size-exclusion chromatography with a Superdex200 10/300 column (GE Healthcare, Chicago, IL, USA). The fractions corresponding to the HA-VLPs peak were pooled and sterile filtered using a Whatman cellulose regenerated membrane filter. In scheme 2, HA-VLPs were purified using a SartoBind Q capsule (Sartorius Stedim Biotech, Göttingen, Germany) according to manufacturer’s instructions. Specifically, elution buffer was composed by HEPES (50 mM) and NaCl (300 mM) at pH 7.4. The fraction corresponding to HA-VLPs was collected and sterile filtered using a Whatman cellulose regenerated membrane filter. Trehalose (pH 7.4) was added to the purified material to a final concentration of 15% (w/v) using a stock solution of 65% (w/v) previously prepared. The resulting purified material was stored at −80 °C (long-term storage) or at 4 °C (short-term storage).

### 2.6. Analytics

#### 2.6.1. Cell Concentration and Viability

Cell counting was performed in a Fuchs–Rosenthal hemocytometer chamber (Brand, Wertheim, Germany) and viability was assessed using the trypan-blue exclusion method.

#### 2.6.2. Hemagglutination Assay

The HA titer was determined using the hemagglutination assay as described elsewhere [26]. Briefly, in-process and purified samples (25 µL) were serially diluted 1:1 with DPBS(-/-) 1X (Gibco, Waltham, MA, USA) in V-bottom 96-well plates (Thermo Scientific, Waltham, MA, USA) and gently mixed 1:1 with 1% chicken erythrocytes (Lohmann, Cuxhaven, Germany). Plates were incubated at 4 °C for 30 min. The HA titer was estimated as being the inverse of the highest dilution of sample that completely inhibited hemagglutination.

#### 2.6.3. SDS-PAGE and Western Blot

In-process and purified samples were denatured by mixing with 1× LDS Sample Buffer (ThermoFisher Scientific, Waltham, MA, USA, stock solution at 4×) and 1× Sample Reducing Agent (ThermoFisher Scientific, Waltham, MA, USA, stock solution at 10×) and heating for 10 min at 70 °C. Denatured samples were loaded into a 4−12% NuPAGE Bis-Tris protein gel (ThermoFisher Scientific, Waltham, MA, USA), using MOPS running buffer (ThermoFisher Scientific, Waltham, MA, USA) and SeeBlue Plus2 Prestained Standard (ThermoFisher Scientific, Waltham, MA, USA) as a molecular weight marker. After electrophoresis (50 min at 200 V and 400 mA), proteins were transferred to a nitrocellulose membrane using the iBlot system (ThermoFisher Scientific, Waltham, MA, USA). Membranes were blocked for 1 h at room temperature with a blocking solution composed of Tris-Buffered Saline (Sigma, St. Louis, MO, USA) with Tween-20 (Millipore, Burlington, MA, USA) and 5% (w/v) skim milk (Millipore, Burlington, MA, USA), and incubated overnight at room temperature with primary antibodies diluted in blocking solution. For HA identification, a mouse monoclonal antibody (IRR, Manassas, VA, USA, FR-494—mouse monoclonal antibody to recombinant H1 HA from influenza A/Brisbane/59/2007 (H1N1)) was used at a dilution of 1:2000. M1 protein was identified using a goat polyclonal antibody (Abcam, Cambridge, UK, Cat# ab20910) at a dilution of 1:2000. Secondary anti-mouse or anti-goat IgG antibodies conjugated with alkaline phosphatase were used at a dilution of 1:2000 for identification of HA and M1, respectively. Protein band detection was performed by covering membranes with NBT/BCIP 1-Step (Thermo Scientific, Waltham, MA, USA) for 10 min; membranes were then scanned with a benchtop scanner device.

#### 2.6.4. Baculovirus Titration

Baculovirus titers were determined using the MTT assay [27,28] (for infectious particles) or qPCR (for total baculovirus genome copies) as described elsewhere [29]. Briefly for qPCR, culture samples were firstly treated with DNAse (Roche, Basel, Switzerland) to eliminate free residual baculovirus DNA prior to quantification. Baculovirus DNA was extracted using the High Pure Viral Nucleic Acid Kit (Roche Life Science, Mannheim, Germany). For the qPCR, a master mix was prepared using the LightCycler 480 SYBR Green I Master (04707516001; Roche Life Science, Mannheim, Germany), a final concentration of 0.5 μM of reverse and forward primers for the ie1 baculovirus gene region, and PCR-grade water. The qPCR was performed in 96-well white plates (04729692001; Roche Life Science, Mannheim, Germany) using a LightCycler 480 Instrument II (Roche Life Science, Mannheim, Germany).

#### 2.6.5. Nanoparticle Tracking Analysis

The concentration and size distribution of HA-VLPs were measured using the NanoSight NS500 (Nanosight Ltd., Salisbury, UK). The samples were pre-diluted with DPBS(-/-) 1X (Gibco, Waltham, MA, USA) to cope with the instrument’s range of analysis (10^8^–10^9^ particles.mL^–1^). All measurements were performed at 22 °C. Sample videos (60 seconds) were analyzed with the Nanoparticle Tracking Analysis (NTA) 2.3 Analytical software. Capture settings (shutter and gain) were adjusted manually.

#### 2.6.6. Transmission Electron Microscopy Analysis

The conformation and size of purified influenza HA-VLPs from bioreactor runs were assessed by negative staining TEM using a Hitachi H-7650 Transmission Electron Microscope (JEOL, Tokyo, Japan). For sample preparation, 10 μL of purified HA-VLPs was fixed for 1 min in a copper grid previously coated with Formvar-carbon (Electron Microscopy Sciences, Hatfield, PA, USA). Grids were then washed three times with water and finally stained with 1% (v/v) uranyl acetate for 2 min. Grids containing fixed samples were left to air dry and immediately analyzed.

### 2.7. Mathematical Equations

#### 2.7.1. Mathematical Equations for Estimation of HA Production Rate

The specific HA production rate, r_HA_, is given by:

(1) $$r_{HA}\left(\mathrm{HA}\;\mathrm{titer}.10^{6}{\;\mathrm{cell}}^{-1}.h^{-1}\right)=\frac{\Delta HA}{\int_{0}^{t}X\;dt}\;,\;0<t<t_{f}\;$$

where HA is the extracellular HA titer (HA titer.mL^−1^) and X is the total cell concentration (10^6^ cell.mL^−1^) during the production phase (from t = 0 to t = t_f_).

#### 2.7.2. Mathematical Equations for Estimation of Reaction Rates

Cell growth rate, μ, is given by:

(2) $$µ\left(h^{-1}\right)=\frac{1}{X}\;.\;\frac{dX}{dt}\;,\;0<t<t_{e}$$

where X is the concentration of viable cells (cell.mL^−1^) and t is the culture time (h) during the exponential growth phase (from t = 0 and t = t_e_).

During the cell growth phase, the specific rates of lactate formation (r_Lac_), and glucose and glutamine consumption (r_Glc_ and r_Gln_), are estimated by:

(3) $$r_{Lac}\left(nmol.10^{6}cell^{-1}.h^{-1}\right)=Y_{X/Lac}\;.\;µ\;$$

(4) $$r_{Glc}\left(nmol.10^{6}cell^{-1}.h^{-1}\right)=Y_{X/Glc}\;.\;µ$$

(5) $$r_{Gln}\left(nmol.10^{6}cell^{-1}.h^{-1}\right)=Y_{X/Gln}\;.\;µ\;$$

where µ is the cell growth rate (h^−1^, as described in Equation (2)), and Y_X/j_ is the yield of metabolite j consumed (Glc or Gln) or produced (Lac) per biomass (X) formed, which is defined as follows:

(6) $$Y_{X/Lac}\left(nmol.10^{6}cell^{-1}\right)=\frac{\Delta \mathrm{Lac}}{\Delta X}\;,\;0<t<t_{e}\;$$

(7) $$Y_{X/Glc}\left(nmol.10^{6}cell^{-1}\right)=\frac{-\Delta \mathrm{Glc}}{\Delta X}\;,\;0<t<t_{e}\;$$

(8) $$Y_{X/Gln}\left(nmol.10^{6}cell^{-1}\right)=\frac{-\Delta \mathrm{Gln}}{\Delta X}\;,\;0<t<t_{e}\;$$

during the exponential growth phase (from t = 0 to t = t_e_).

During the production phase (i.e., infection), the yield of product (HA) formed per substrate (Glc or Gln) consumed is defined as follows:

(9) $$Y_{Glc/HA}\left(HA\;titer.nmol^{-1}\right)=\frac{\Delta \mathrm{HA}}{-\Delta \mathrm{Glc}}\;,\;0<t<t_{f}\;$$

(10) $$Y_{Gln/HA}\left(HA\;titer.nmol^{-1}\right)=\frac{\Delta \mathrm{HA}}{-\Delta \mathrm{Gln}}\;,\;0<t<t_{f}$$

where HA is the extracellular HA titer (HA titer.mL^−1^), Glc is the concentration of glucose (mM) and Gln is the concentration of glutamine (mM) during the production phase (from t = 0 to t = t_f_).

### 2.8. Statistical Analysis

Data were expressed as the mean ± standard deviation from three independent biological replicates. Differences in cell-specific HA productivity between adapted and non-adapted cells were tested by a one-way ANOVA using Dunnett’s multiple comparison analysis method (adjusted *p*-value < 0.05 was considered statistically significant). Pearson’s correlation (r) was used to analyze the similarity between the shake-flask and stirred-tank bioreactor (r = 1 representing the best linear fit between the data) in terms of extracellular HA titer.

## 3. Results

### 3.1. Increased Production of Influenza HA-Vlps in Adapted Cells

**Figure 1:**
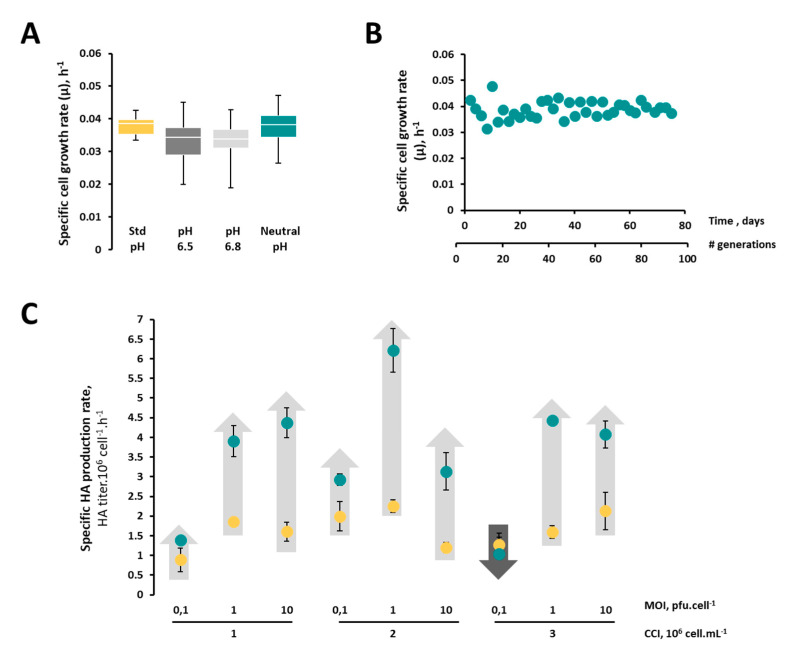
Adaptation of High Five insect cells to neutral culture pH for influenza hemagglutinin (HA)-displaying virus-like particle (VLP) production. (**A**) Box-plot diagrams showing the degree of dispersion and skewness in specific cell growth rate (µ, h^−1^) values during the adaptation process; horizontal lines are medians, boxes represent the interquartile range, and error bars show the full range of values. (**B**) Scatter graph representing the variation in growth rate of adapted cells over 100 generations after establishment of master cell bank. (**C**) Specific HA production rate of adapted (green circles) and non-adapted (yellow circles) cells at different cell concentration at the time of infection (CCI)/multiplicity of infection (MOI) combinations; arrows represent the variation in specific HA production rates from non-adapted to adapted cells, where light grey denotes an increase and dark grey denotes a decrease (cell-specific production rate was calculated as described in M&M section)—data represent one biological replicate (n = 1).

Aiming at improving the production yields of influenza HA-VLPs, High Five insect cells were adapted to grow at a neutral culture pH (7.0) instead of a standard culture pH (6.2). Given the significant difference between the standard and neutral pH, a single step change in culture pH resulted in a loss of cell viability and incapacity to maintain cells in culture (data not shown). Thus, a stepwise approach was used for the adaptation process, in which the culture pH was gradually increased from standard 6.2 to 6.5, 6.8 and finally to 7.0. Upon each change in culture pH, a decrease in cell viability and growth rate was observed, but standard values were quickly re-established. Adaptation to each culture pH endured for 2–3 weeks. In approximately 8 weeks, cells were fully adapted to grow at a neutral pH, showing a growth rate similar to that of non-adapted cells cultured at a standard pH (Figure 1A). Importantly, adapted cells were able to maintain their growth rate (≈0.04 h^−1^, i.e., ≈18 h per generation) and viability for more than 100 generations (Figure 1B), thus demonstrating their stability when routinely cultured in such a non-physiological condition (i.e., neutral pH).

The capacity of adapted cells to produce HA-VLPs was evaluated at different combinations of MOIs (0.1, 1 and 10 pfu.cell^−1^) and CCIs (1, 2 and 3 × 10^6^ cell.mL^−1^), and compared to that of non-adapted cells. Adapted cells performed better than their non-adapted counterparts in all combinations tested as depicted by an increase in specific productivity (Figure 1C), the exception being the combination MOI of 0.1 pfu.cell^−1^ and CCI of 3 × 10^6^ cell.mL^−1^. The best infection condition was achieved using a CCI of 2 × 10^6^ cell.mL^−1^ and MOI of 1 pfu.cell^−1^ as it maximized the cell-specific HA production rate (6.2 HA titer.10^6^ cell^−1^.h^−1^) concomitantly with the HA titer (768 HA titer.mL^−1^) (see Figure S1). The specific HA productivity of adapted cells was similar at different generations after the establishment of the master cell bank using the best infection condition (6.7 ± 0.6, 5.3 ± 0.5 and 5.8 ± 0.5 HA titer.10^6^ cell^−1^.h^−1^ at generations 10, 40 and 80, respectively). These infection parameters were used in subsequent studies for the characterization of the adapted cell population.

### 3.2. The Adaptation Process (and Not pH Shift) Is the Element Driving Increased Specific HA Productivity

To evaluate if a pH shift at the time of infection is sufficient to increase HA production in non-adapted cells, these were infected either in standard pH 6.2 culture medium (CM_pH6.2_) or in neutral pH culture medium (CM_pH7_). Similarly, adapted cells were infected either in CM_pH7_ or in CM_pH6.2_.

**Figure 2:**
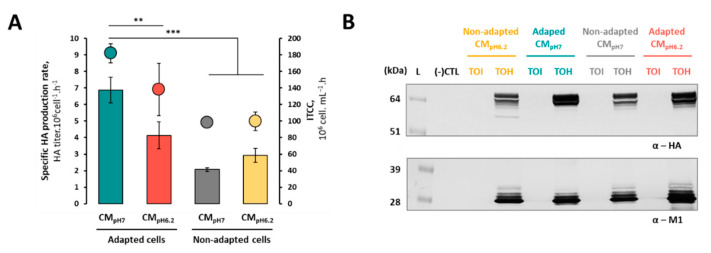
Impact of pH shift on HA production. (**A**) Bars represent specific HA productivity (HA titer.10^6^ cell^−1^.h^−1^) and dots represent total number of cells during production phase (integral of total cell concentration, ITCC, 10^6^ cell.mL^−1^.h) of adapted and non-adapted cells in neutral pH culture medium (CM_pH7_) or in standard pH 6.2 (CM_pH6.2_); (**B**) identification of HA and M1 proteins by Western blot; samples were collected at the time-of-infection (TOI) and time-of-harvest (TOH) for adapted and non-adapted cells in neutral pH culture medium (CM_pH7_) or in standard pH 6.2 (CM_pH6.2_). Samples loaded are clarified supernatants, non-diluted; same volume was loaded in the gel for all samples. Relative band intensities for TOH samples (overproduction with non-adapted cells in CM_pH6.2_) are 1.5:0.8:1.6 (for HA) and 1.1:0.8:1.5 (for M1) for adapted cells-CM_pH7_: non-adapted cells-CM_pH7_: adapted cells-CM_pH6.2_, respectively. Uncropped membranes can be observed in Supplementary Figure S2. For Figure (**A**): data are relative to three biological replicates (n = 3); differences were tested with one-way ANOVA using Dunnett’s multiple comparisons test (** = adjusted *p*-value < 0.005 and *** = adjusted p-value < 0.001 were considered statistically significant). For Figure (**B**): (-) CTL denotes negative control (supernatant of non-infected cells) and L denotes pre-stained protein standard SeeBlue^®^ Plus2.

Non-adapted cells performed similarly when producing in CM_pH7_ and CM_pH6.2_ (Figure 2A), meaning that a pH shift to a neutral pH upon infection does not suffice for improving specific HA productivity. In fact, a pH shift at the time of infection can even be detrimental for HA production, as exemplified for adapted cells, with a 40% reduction in the cell-specific HA production rate and 25% decrease in the total number of cells during the production phase (i.e., integral of total cell concentration, ITCC) when infection of these cells was performed in CM_pH6.2_ rather than their optimal CM_pH7.0_. Overall, cell-specific HA productivity was higher in adapted cells when compared to non-adapted cells, regardless of the culture medium pH. A Western blot analysis corroborates these findings, with HA protein expression at the time of harvest (i.e., at cell viability ≈ 30–45%) being superior in adapted cells (Figure 2B) as compared to non-adapted cells. These results demonstrate that the adaptation process, rather than the pH shift at the time of infection, is essential to achieve a higher specific HA productivity.

### 3.3. Delayed Cell Lysis and Higher Specific HA Production Rate of Neutral pH Adapted Cells

To investigate the impact of the adaptation process on infection kinetics (including baculovirus replication) and HA expression, adapted and non-adapted cells were cultured in their respective media (CM_pH7_ and CM_pH6.2_, respectively) and infected under the best conditions identified above (CCI of 2 × 10^6^ cell.mL^−1^ and MOI of 1 pfu.cell^−1^).

**Figure 3:**
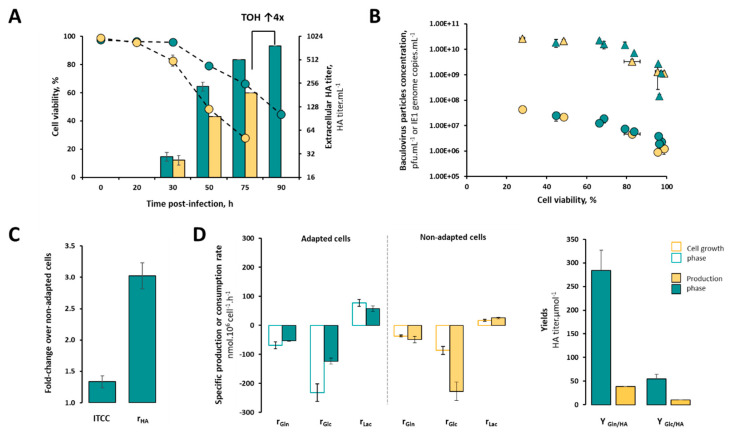
Infection kinetics, HA expression and cell metabolism read-outs of adapted and non-adapted High Five cells during production of influenza VLPs. (**A**) Cell viability (%) and extracellular HA titer (HA titer.mL^−1^) profile upon infection; (**B**) infectious (circles, pfu.mL^−1^) and total (triangles, IE1 genome copies.mL^−1^) baculovirus titers upon infection; (**C**) fold-change (adapted/non-adapted) of total number of cells during production phase (ITCC, 10^6^ cell.mL^−1^.h) and specific HA productivity (r_HA_, HA titer.cell^−1^.h^−1^); (**D**) specific consumption rate of glucose (r_Glc_, nmol.10^6^ cell^−1^.h^−1^) and glutamine (r_Gln_, nmol.10^6^ cell^−1^.h^−1^), specific production rate of lactate (r_Lac_, nmol.10^6^ cell^−1^.h^−1^), and yields of product (HA) formed per substrate (Glc or Gln) consumed; i.e., Y_Glc/HA_ and Y_Gln/HA_. Color code: green represents data for adapted cells, and yellow represents data for non-adapted cells. Data are expressed as mean ± standard deviation (relative to three biological replicates, n = 3).

Differences in infection kinetics (i.e., drop in cell viability) and HA expression (i.e., extracellular HA titer) were observed (Figure 3A). Adapted cells maintained higher cell viability values throughout the course of infection in comparison with non-adapted cells, delaying the onset of the cell viability drop by 20–40 h. In addition, extracellular HA titers are higher in adapted cells irrespective of the culture time; at the time of harvest, this difference reaches ≈ 4-fold. To ensure that titers are not being overestimated by the presence of baculovirus particles carrying HA in culture medium, infectious and total baculovirus titers were assessed (Figure 3B). The increase in infectious baculovirus titers (pfu.mL^−1^) as cell viability decreases throughout the infection is similar in adapted and non-adapted cells. The same pattern was observed for total baculovirus particles (IE1 genome copies.mL^−1^), thus suggesting that the increased extracellular HA titer obtained with adapted cells does not derive from baculovirus particles carrying HA but rather from the synergistic effect of a higher total number of cells during the production phase—i.e., ITCC, 10^6^ cell.mL^−1^.h (≈ 1.34-fold)—and specific HA productivity, i.e., r_HA_, HA titer.cell^−1^.h^−1^ (≈ 3-fold) (Figure 3C).

The consumption of glucose (Glc) and glutamine (Gln), and the production of lactate (Lac) were followed throughout the culture, and their specific consumption ($r_{Glc}$ and $r_{Gln}$) or production ($r_{Lac}$) rates were estimated accordingly (Figure 3D).

During the growth phase, the $r_{Glc}\;$(nmol.10^6^ cell^−1^.h^−1^) and $r_{Gln}$ (nmol.10^6^ cell^−1^.h^−1^) of adapted cells were 2.7- and 2.0-fold higher than those of non-adapted cells. Likewise, lactate was also produced at a higher rate in adapted cells; nonetheless, this byproduct did not accumulate growth-impairing values as the concentration achieved was ≈ 3-fold lower than that reported in the literature as the maximum for insect cells (15 mM [30]).

Upon infection, $r_{Glc}$, $r_{Gln}$ and $r_{Lac}$ changed in both adapted and non-adapted cells, with a reduction in all rates in adapted cells contrasting with an increase in non-adapted cells.

These differences in cell metabolism reflect a more efficient utilization of carbon and nitrogen sources for product formation in adapted cells as suggested by the higher yields obtained (i.e., Y_Glc/HA_ ≈ 5 and Y_Gln/HA_ ≈ 7-fold).

### 3.4. Proof-of-Concept at the 0.5 L Bioreactor Scale

The feasibility of adapted cells to produce influenza HA-VLPs in a computer-controlled and scalable system was confirmed in 0.5 L stirred-tank bioreactors (STBs). Adapted cells at generation 53 were infected in STBs and shake flasks (SFs) using previously optimized conditions (CCI = 2 × 10^6^ cell.mL^−1^ and MOI = 1 pfu.cell^−1^); the infection kinetics and HA titer obtained in both culture systems were compared.

**Figure 4:**
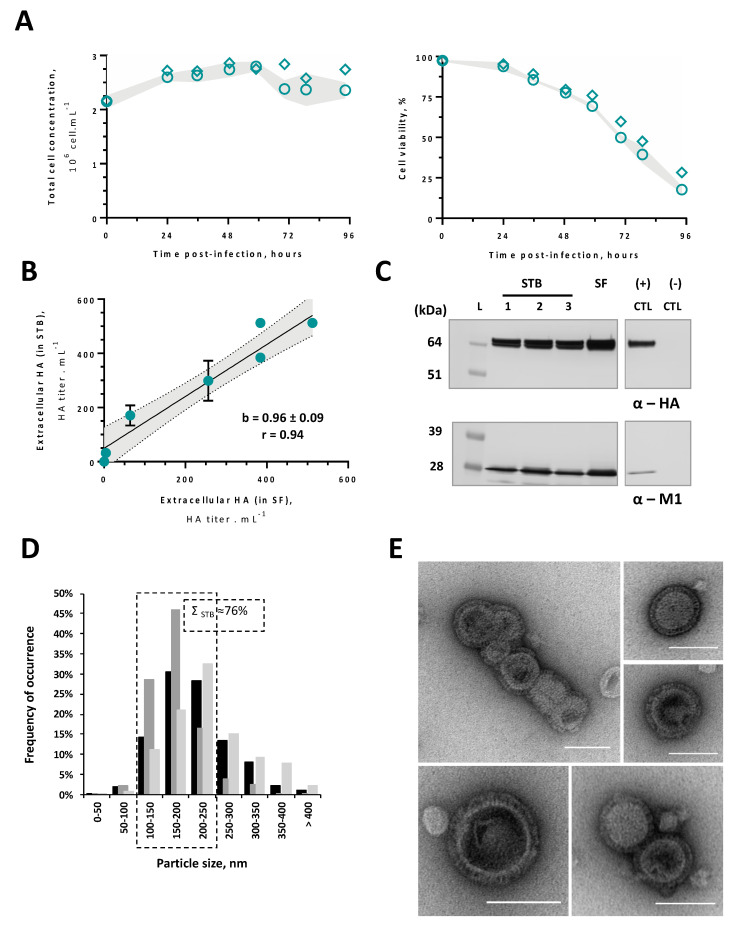
Production of influenza VLPs at a 0.5 L bioreactor scale. (**A**) Cell growth and viability kinetics upon infection in three stirred-tank bioreactors (STBs, circles) and one control shake flask (SF, diamonds), with the grey band representing standard deviation (relative to three biological replicates, n = 3) from STB cultures; (**B**) comparison of extracellular HA titers throughout infection between STB and SF, with straight line representing the best linear fit to the data (r—Pearson’s correlation, m—regression slope) and the grey band representing a 95% confidence level. Data are expressed as mean ± standard deviation (relative to three biological replicates, n = 3) from STB cultures. (**C**) identification of HA and M1 proteins by Western blot in in-process samples at the time of harvest from three STB cultures and one control SF culture. Samples loaded are clarified supernatants, non-diluted; same volume was loaded in the gel for all samples. (+) CTL (positive control): master virus seed stock used for influenza VLPs production. (-) CTL (negative control): supernatant of non-infected cell culture. L: Pre-stained protein standard SeeBlue^®^ Plus2; Relative band intensities (overproduction in SF) for STB1: STB2: STB3 are 0.8: 0.9: 0.9 (for HA) and 0.8: 1.1: 0.8 (for M1). Uncropped membranes can be observed in Supplementary Figure S3. (**D**) Histogram showing the size distribution profile of HA-VLPs obtained by nanoparticle tracking analysis (binned by 50 nm intervals from 0 to >400 nm) in purified HA-VLP samples. Overlapping bars represent the three bioreactor runs. The average percentage of HA-VLPs in the expected size range of 100–250 nm is highlighted; (**E**) negative-staining transmission electron microscopy of purified HA-VLPs. Scale bar represents 100 nm.

Cell growth and viability kinetics in STBs and SFs were similar, showing traditional profiles of insect cells upon infection with an MOI of 1 pfu.cell^−1^ (Figure 4A). Likewise, extracellular HA titers throughout infection were similar between STBs and SFs, with a regression coefficient (b) and Pearson’s correlation (r) close to 1 (Figure 4B). Both HA and M1 proteins could be identified at the time-of-harvest in supernatant samples from STB and SF cultures by Western blot (Figure 4C), showing similar level of HA expression. Influenza HA-VLPs produced in STB were concentrated and purified as mentioned in M&M, and then analyzed by nanoparticle tracking analysis (Figure 4D) and negative staining TEM (Figure 4E). A normalized frequency histogram of size distribution confirms the presence of a single peak corresponding to the normal-size range of M1-HA-VLPs (≈ 100–250 nm) (Figure 4D). The presence of particles resembling HA-VLPs, both in size and morphology, was confirmed by TEM (Figure 4E). These results confirm the scalability of the strategy herein proposed (i.e., High Five insect cells adapted to grow at neutral culture pH instead of standard pH 6.2) for the production of influenza HA-VLPs.

## 4. Discussion

In this work, we adapted the High Five insect cell line to grow at neutral culture pH (7.0) and evaluated its potential to be used for the production of influenza HA-VLPs.

High Five cells were efficiently adapted to grow at neutral culture pH (7.0), showing similar specific growth rates (≈ 0.04 h^−1^) and morphologic characteristics (round shape with diameter ≈ 16–17 µm [31,32]) to non-adapted cells. In addition, adapted cells were efficiently maintained in culture for over 100 population doublings, thus demonstrating their stability to be routinely cultured in such a non-standard condition. The capacity of these cells for the improved production of HA-VLPs was demonstrated at different combinations of CCIs and MOIs, with CCI = 2 × 10^6^ cell.mL^−1^ and MOI = 1 pfu.cell^−1^ maximizing the specific HA production rate (6.2 HA titer.10^6^ cell^−1^.h^−1^) and extracellular HA titer (768 HA titer.mL^−1^). Under this optimal condition, adapted cells not only expressed more protein (up to 4-fold higher HA titer.mL^−1^) but also showed a delayed onset of cell viability drop (up to 20–40 h) when compared to non-adapted cells, thus enabling (i) harvesting the same amount of HA protein at a higher cell viability, which facilitates downstream processing and prevents HA degradation by proteases, or (ii) harvesting significantly higher amounts of HA proteins, which reduces working volumes and increases production load. Further process optimization may be possible through the enrichment of an adaptation medium in essential nutrients (e.g., glucose and glutamine) to values similar to those of a standard medium, given that adapted cells show a more efficient utilization of carbon and nitrogen sources for product formation.

Shifts in culture parameters to non-physiological values have been shown to impact the growth performance and recombinant protein production yields of prokaryotes [33,34,35] and other higher level organisms [10,11,14,36,37]. In this study, shifting the culture pH from standard 6.2 to 7.0 was not sufficient for improving HA production in parental, non-adapted cells. In adapted cells, a pH shift (from 7.0 to standard 6.2) was even detrimental for HA production. Osmolarity could possibly play a role on productivity [38]. However, our data show that culture media at pH 6.2 and 7.0 have similar osmolarities; i.e., 344 and 363 mOsm, respectively, suggesting this is not contributing to the higher productivity of adapted cells. Therefore, it is possible to conclude that the adaptation process is essential to improve productivity and that infection at a neutral pH is required to maximize titers. Adaptation rather than culture parameter shifts have also been reported elsewhere as necessary to improve the production of recombinant proteins [23,24,39].

The production of HA-VLPs using adapted cells was scaled-up from the shake flask (SF) to the 0.5 L stirred-tank bioreactor (STB). Cell growth and HA production kinetics were similar between SFs and STBs, thus resulting in similar specific production rates. HA and M1 proteins were identified in both STB and SF cultures. The TEM analysis of purified HA-VLPs revealed a multi-component organization and the presence of HA molecules (spikes) uniformly displayed on VLPs’ surfaces, similar to HA-VLPs shown in other reports [40]. Overall, these results confirm (i) the null impact of adaptation process and neutral culture pH on the quality of HA-VLPs produced, and (ii) the feasibility of the strategy herein proposed (i.e., adaptation of High Five cells to neutral culture pH) for an improved production of HA-VLPs.

The mechanism(s) underlying the higher capacity of neutral pH-adapted cells to produce HA remain(s) unclear. Importantly, the proteolytic activity of each particular cell or viral protease varies differently depending on culture pH, potentially involving a loss of the product of interest [41]. Moreover, it has been shown that changes in the extracellular pH of mammalian cell cultures induces fluctuations in intracellular pH [42], with varying impacts on cell metabolism [43], enzyme activity [41] and protein synthesis [44]. Unlike mammalian cells, insect *Sf*-9 cells have a strong buffering capacity, maintaining intracellular pH at a neutral value even upon variations in culture pH of up to 6.8 (from standard culture medium pH 6.2) or infection with recombinant baculoviruses [45]. Assessing the intracellular pH of adapted and non-adapted cells during growth and HA-VLPs production would clarify if these values differ and, if so, help dissect its impact on cell behavior including metabolism and enzyme activity (cellular and viral) and thus protein production. Regardless, the smaller difference between the extracellular and intracellular pH of adapted cells could possibly result in less energy waste to maintain homeostasis, thus benefiting recombinant protein production.

Different cell lines and/or expression systems have been employed for the production of influenza HA-VLPs, yielding 128 HA titer.mL^−1^ [26], 16 HA titer.mL^−1^ [9], or even above 300 HA titer.mL^−1^ [46,47,48]. Noteworthy, the production process herein developed was shown to yield an extracellular HA titer similar to those reported when using non-adapted cells (192 HA titer.mL^−1^) or higher when using adapted cells (768 HA titer.mL^−1^). Regardless, the process yield can be significantly influenced by factors such as the culture medium, quality of virus stock, or even the HA strain being produced.

## 5. Conclusions

This work demonstrates the use of ALE as a valuable approach to increase the production yields of influenza HA-VLPs in High Five insect cells. A new cell population efficiently adapted to grow at a neutral pH, which induced higher cell-specific HA productivity and extracellular HA titers, and a showed delayed onset of the cell viability drop, thus enabling users to anticipate harvesting or to harvest larger amounts of protein. It is noteworthy that the adaptation process (and not pH shift) is essential for increased specific HA productivity.

To our knowledge, this is the first report exploring ALE in *Trichoplusia ni* insect cell lines and its impact on the production of an influenza vaccine candidate. The work here encourages the use of ALE as an alternative bioprocess strategy to improve production yields of other insect-derived biologics.

## Supplementary Materials

The following are available online at https://www.mdpi.com/2076-393X/8/4/589/s1. Figure S1: Extracellular HA titer (HA titer.mL^−1^) of adapted cells (dark grey) and non-adapted cells (light grey) at different CCI/MOI combinations. Arrows and symbols next to each graph represent the variations in HA titer at the time-of-harvest from non-adapted to adapted cells for each CCI/MOI combination; Figure S2: Identification of HA and M1 proteins by Western blot; in-process samples were collected at the time-of-infection (TOI) and time-of-harvest (TOH) for adapted and non-adapted cells in neutral pH culture medium (CM_pH7_) or in standard pH 6.2 (CM_pH6.2_). Samples loaded are clarified supernatants, non-diluted; same volume was loaded in the gel for all samples. Band intensities for TOH samples are shown and are normalized to production performed with non-adapted cells in CM_pH6.2_; Figure S3: Identification of HA and M1 proteins by Western blot in in-process samples at the time of harvest from three STB cultures and one control SF culture. Samples loaded are clarified supernatants, non-diluted; same volume was loaded in the gel for all samples. (+) CTL (positive control): master virus seed stock used for influenza VLPs production. (-) CTL (negative control): supernatant of non-infected cell culture. L: Pre-stained protein standard SeeBlue^®^ Plus2. Band intensities are normalized to production performed in SF.
