# Supplementary material for: Improving Influenza HA-Vlps Production in Insect High Five Cells via Adaptive Laboratory Evolution
Source: Vaccines (Basel). 2020 Oct 7;8(4):589. doi: 10.3390/vaccines8040589 (PMC7711658; doi:10.3390/vaccines8040589)
Supplement: Supplementary file 1 [file vaccines-08-00589-s001.pdf]

vaccines

Supplementary Material

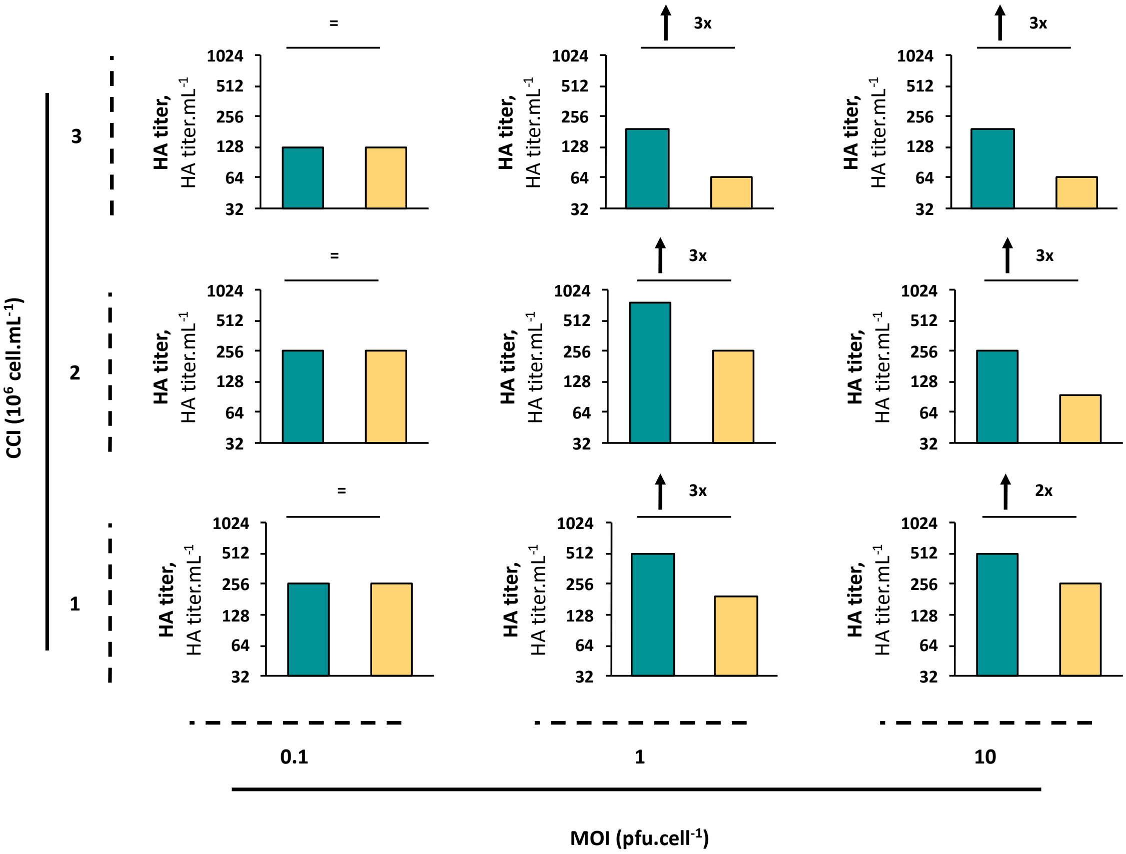

Figure S1. Extracellular HA titer (HA titer. mL^{-1}) of adapted cells (dark grey) and non-adapted cells (light grey) at different CCI/MOI combinations. Arrows and symbols next to each graph represent the variations in HA titer at the time-of-harvest from non-adapted to adapted cells for each CCI/MOI combination.

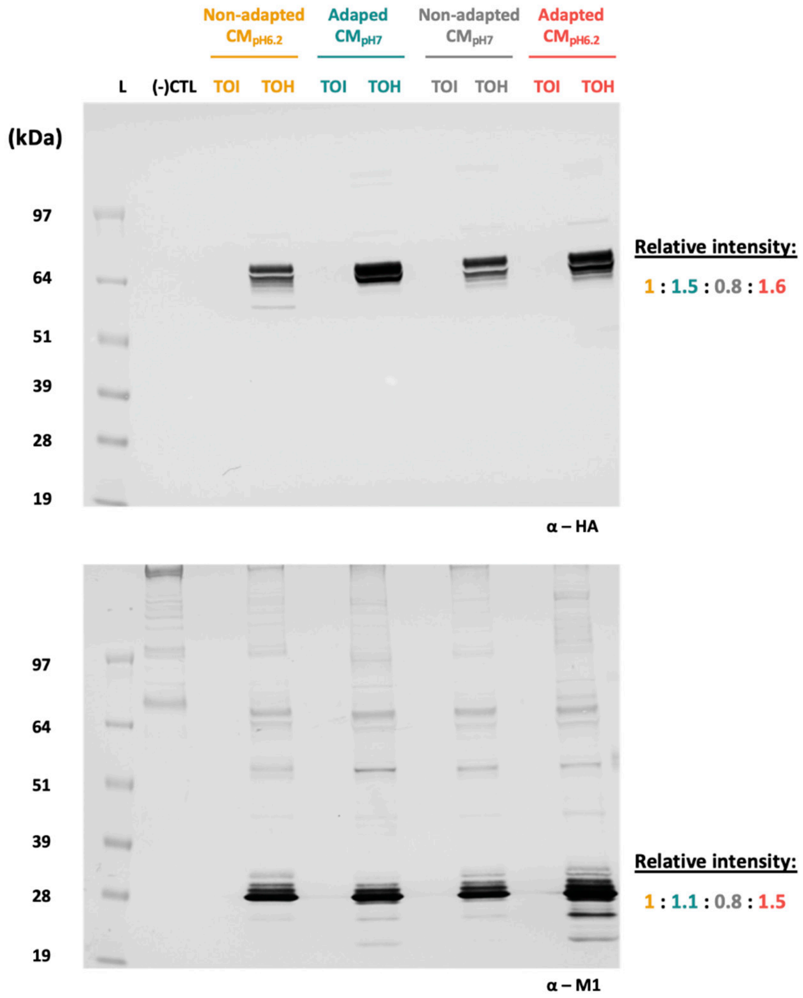

Figure S2. Identification of HA and M1 proteins by western blot; In-process samples were collected at the time-of-infection (TOI) and time-of-harvest (TOH) for adapted and non-adapted cells in neutral pH culture medium (CM_{pH7}) or in standard pH 6.2 ($CM_{pH6.2}$). Samples loaded are clarified supernatants, non-diluted; same volume was loaded in the gel for all samples. Band intensities for TOH samples are shown and are normalized to production performed with non-adapted cells in $CM_{pH6.2}$.

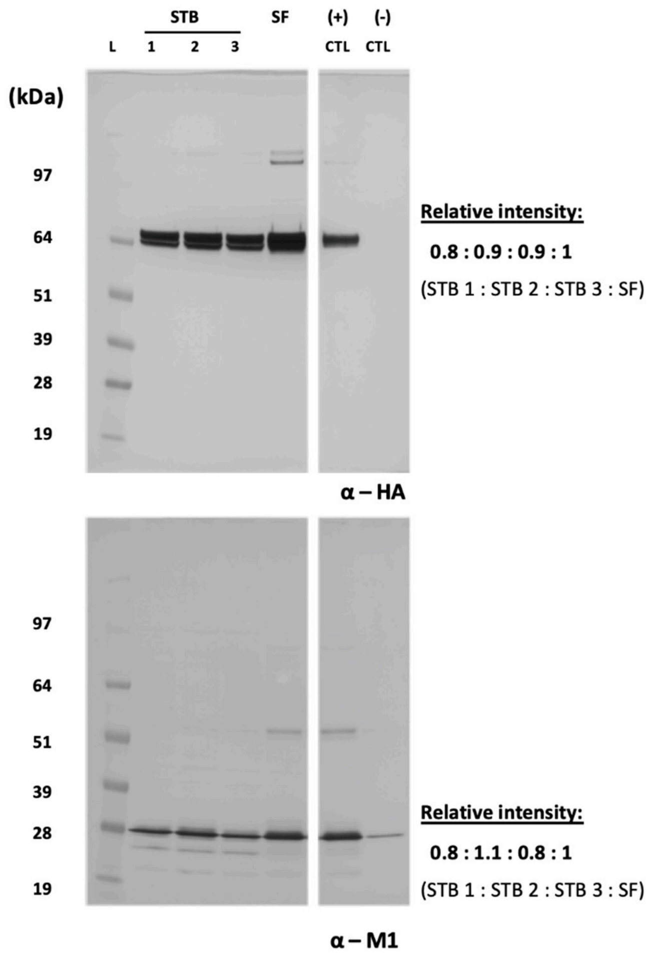

Figure S3. Identification of HA and M1 proteins by Western blot in in-process samples at the time of harvest from three STB cultures and one control SF culture. Samples loaded are clarified supernatants, non-diluted; same volume was loaded in the gel for all samples. (+) CTL (positive control): master virus seed stock used for influenza VLPs production. (-) CTL (negative control): supernatant of non-infected cell culture. L: Pre-stained protein standard SeeBlue® Plus2. Band intensities are normalized to production performed in SF.
